# Rotavirus Infection and Genotyping in Yantai, Shandong Province, 2017–2019

**DOI:** 10.3390/tropicalmed8020101

**Published:** 2023-02-03

**Authors:** Zhenlu Sun, Guifang Zhang, Chunyan Li, Peihua Niu, Xia Li, Qiao Gao, Kai Guo, Ruiqing Zhang, Ji Wang, Xuejun Ma

**Affiliations:** 1 Yantai Center for Disease Control and Prevention, Yantai 264003, China; 2 NHC Key Laboratory of Medical Virology and Viral Diseases, National Institute for Viral Disease Control and Prevention, Chinese Center for Disease Control and Prevention, No. 155, Changbai Street, Changping District, Beijing 102206, China; 3 Center for Biosafety Mega-Science, Chinese Academy of Sciences, Wuhan 430071, China

**Keywords:** diarrhea, serotypes, genotyping, rotavirus, vaccine, infection

## Abstract

Purpose: Rotavirus (RV) ranked first among infectious diarrhea-causing pathogens in Yantai from 2017 to 2019. This study investigated the seroserotypes of RV in Yantai, Shandong, from 2017 to 2019 to identify the dominant serotypes and explore the epidemic pattern, aiming to effectively reduce the infection rate, better guide vaccination, and help in epidemiological prevention and control. Methods: A total of 2227 human diarrhea samples were collected from 2017 to 2019 in Yantai. The VP7 (G serotype) and VP4 (P serotype) genes of 467 RV-positive samples were amplified using two-round nested reverse transcription–polymerase chain reaction for G/P genotyping. Results: The genotyping results of RV in Yantai from 2017 to 2019 revealed that G9 was the dominant serotype for all G serotypes, P[8] was the dominant serotype for all P serotypes, and G9P[8] was the dominant serotype for all G/P combinations. G9 serotype accounted for 60.84%, 95.65%, and 83.76% of the total RV samples collected in 2017, 2018, and 2019, respectively. P[8] accounted for 75.52%, 94.69%, and 88.89% of the RV-positive samples collected in 2017, 2018, and 2019, respectively. G9P[8] accounted for 60.84%, 94.69%, and 83.76% of the total RV samples collected in 2017, 2018, and 2019, respectively. Of the total 467 samples from 2017 to 2019, G2P[4] accounted for 3.64% (17/467), G3P[8] for 1.28% (6/467), and G1P[8] for 0.86% (4/467). Conclusion: This study revealed the epidemiological characteristics of RV infection and the development pattern of dominant serotypes in Yantai in recent years, guiding the selection of RV vaccines. The prioritization of vaccines containing G9 serotype for infants in Yantai in recent years is recommended.

## 1. Introduction

Rotavirus (RV), with a spherical shape and size of 70 nm, belongs to the family reoviridae and is a nonenveloped RNA virus. It is composed of double-stranded RNA, which contains 11 genomes and is surrounded by a three-layer icosahedral protein shell. Each genome segment encodes the proteins with various functions. The outer proteins (viral proteins VP4 and VP7) mediate attachment and penetration. The inner layer consists of VP2 and is surrounded by the viral genome and minor structural protein VP1; moreover, it depends on the RNA polymerase of viral RNA and VP3. The middle layer comprises VP6, which interacts with proteins and stabilizes the inner and outer layers [1]. VP6 can be used to identify RV serotypes [2]. Of the RVs, the viral structural protein is VP1–VP7, and the nonstructural protein is NSP1–NSP6. The RV gene 11 encodes NSP5 and NSP6 [2,3,4]. RV can be distinguished using a dual classification system based on two outer capsid proteins: VP4 defines P (protease-sensitive) and VP7 defines G (glycoprotein), that is, the seroserotype of the virus. The surface proteins VP7 and VP4 can independently induce neutralizing antibodies in vivo, making them sites for vaccine development [5]. So far, at least 36 G serotypes and 51 P serotypes have been identified in humans and animals [6].

RV is an important pathogen inducing infectious diarrhea in adults and children, especially infants. RVs are classified into A–G serotypes based on antigenic differences. The most common RV in humans is the group A rotavirus (RVA), which is the target serotype of the current vaccination program. Except for RVA, group B, C, and H rotaviruses are also associated with gastroenteritis in humans. Especially group B, which is also known as an adult diarrhea rotavirus, can cause severe diarrhea in adults. The group C rotavirus mainly infects children aged 4–7 years, and each epidemic is sporadic and self-limited; however, group D, E, and G rotaviruses only infect poultry [7].

RV causes rotavirus gastroenteritis (RVGE) in infants and children mainly through the fecal–oral route. It also causes dyspepsia and diarrhea by attacking and damaging the mucosal cells of the mature epithelium of the small intestine. RV can also attack other organ tissues outside the intestinal tract and cause damage. RV can also attack other organ tissues outside the intestine and cause damage. Almost every child has been infected with RV before the age of 5 years. Among infants and children younger than 5 years of age, RV-related hospitalized cases account for 32–50% of all hospitalized patients with diarrhea [8]. Fecal–oral transmission is the main route of transmission. The course of the disease generally spans 6–7 days, with severe cases presenting dehydration. The RV detection rate is similar in developed and developing countries. According to statistics, RV is the main cause of death due to diarrhea in children, posing a large economic burden to families and society [9,10,11,12]. There are no specific drugs available for rotavirus diarrhea, and it can only be prevented by vaccines. Therefore, the development of a safe and efficient vaccine is important to prevent and control rotavirus disease. Moreover, the development of safe and efficient vaccines is important to prevent and control the occurrence of rotavirus diseases and reduce economic losses. Vaccination is the most effective means to prevent RV infection currently. In 2006, the World Health Organization recommended promoting RV vaccination in high- and middle-income countries [13].

Various subserotypes of rotavirus may exist in different regions. The continuous and comprehensive surveillance of the serotypes in an area to identify local dominant serotypes is beneficial for selecting appropriate vaccine components for better prevention as it can more effectively reduce RV infection rates as part of a comprehensive approach prevent and treat diarrhea. It is also important to understand the distribution of rotavirus genotypes for targeted research and the development of rotavirus vaccines. Continued monitoring of rotavirus genotypes transmitted globally will provide important data for epidemiology and genotype diversity and provide a reference for selecting appropriate genotypes for current and future vaccine development. Therefore, this study investigated the RV serotypes in Yantai, Shandong, from 2017 to 2019 to explore its epidemic pattern and better guide vaccination and epidemiological prevention and control.

## 2. Materials and Methods

### 2.1. Sample Source 

The clinical and laboratory diagnoses of infectious diarrhea were carried out according to the Diagnostic Criteria for Infections Diarrhea (WS271-2007) [14]. When a suspected case of infectious diarrhea was found in the 13 districts and counties of Yantai, the medical institutions at all levels conducted direct online reporting, following the Infectious Disease Reporting and Regulations. Stool specimens were collected from all patients with diarrhea within 3 days of onset. Each specimen was stored at −20 °C for 3–5 mL, and the “Diarrhea Specimen Registration Form” was filled out. The district CDC sent the specimens and related forms collected in the previous month to the Yantai CDC by the 10th of each month.

### 2.2. Sample Collection

The fecal samples were collected from 2227 patients with clinical infectious diarrhea in Yantai from 2017 to 2019. For this, 3–5 g of each sample was taken and placed in a sterile, dry collection tube (preferably not a glass container). Protective agents, culture media, and so forth were not added to the container in advance. The fecal samples were diluted in advance, and anal swabs were collected as much as possible. No antibiotics were taken before sampling. After collection, the samples were refrigerated at 4 °C, sent to the laboratory within 24 h, and frozen at −80 °C for centralized detection. Repeated freezing and thawing was avoided.

A total of 467 fecal treatment fluids with positive RV in diarrhea surveillance samples were selected from 2017 to 2019 in Yantai for G/P genotyping.

### 2.3. Extraction of Viral RNA

The frozen fecal samples were dissolved, and 0.1 mL of the liquid fecal sample and 0.9 mL of normal saline were added to a 1.5-mL Eppendorf tube to prepare 10% fecal suspension, vortexed three times, and centrifuged at 3000 rpm for 5 min at room temperature. The supernatant was taken for nucleic acid extraction [15]. A QIAamp Viral RNA Mini Kit (QIAGEN GmbH, GERMANY) was used to extract RNA following the manufacturer’s protocols, strictly following the reagent operation instructions and our interpretation of the results. All samples were tested on the same day with quality control in place. The reagents used were within the validity period.

### 2.4. G/P Typing of Rotavirus

For RV-positive specimens, nested two-round RT-PCR was used to amplify VP7 (G typing) and VP4 (P typing) gene regions for genotyping. Primers (Table 1, Table 2, Table 3 and Table 4) and amplification conditions were shown in reference [16]. Electrophoresis and digital generation analysis were performed using the QIAGEN OneStep RT-PCR Kit (QIAGEN GmbH, GERMANY) and the QIAxcel capillary electrophoresis apparatus of Genstar Conrun Bio’s 2xTaq PCR Starl MiX. G and P genoserotypes (Genstar Biochem, Canada) were determined according to the size of specific nucleic acid bands.

After the PCR, 10 μL of the product was analyzed using agarose gel electrophoresis (2%), and G and P genoserotypes were determined according to the size of specific nucleic acid bands.

### 2.5. Statistical Processing

Excel 2007 was used to organize and graph the case information data. Excel 2007 was used to organize and graph the data, and data processing and statistical analysis were performed using SPSS17.0 statistical software for data processing and statistical analysis; moreover, the count data were expressed as rates, and the χ^2^ test was used for comparison between groups. The χ^2^ test was used for comparison between groups, and *p* < 0.05 indicated that the difference was statistically significant.

## 3. Results

### 3.1. Crowd Distribution

From 1 January 2017 to 31 December 2019, a total of 467 rotavirus positives were detected in diarrhea surveillance samples in Yantai, Shandong Province, with subjects aged 0–88 years. The results of the study showed that rotavirus infection was mainly concentrated in infants and children aged 0–5 years, and the positivity rate was 69.25%. There were 220 males and 247 females, and the results showed a lower ratio among male patients than female patients; moreover, the difference was statistically significant (χ^2^ = 15.218, *p* < 0.05).

### 3.2. Time Distribution

The seasonal distribution pattern of the rotavirus for three consecutive years from 2017 to 2019 was basically the same, with cases reported in all months, with obvious seasonal distribution characteristics, and the peak of infection mainly occurred in winter and spring. In most years, rotavirus infection cases began to increase significantly after October, peaked in November or December of that year, and the incidence began to decline in April of the following year, with the lowest infection rate in June–August of each year.

### 3.3. Electrophoresis Result of G/P Genotyping of RV in Some Samples from 2017 to 2019

**Figure 1:**
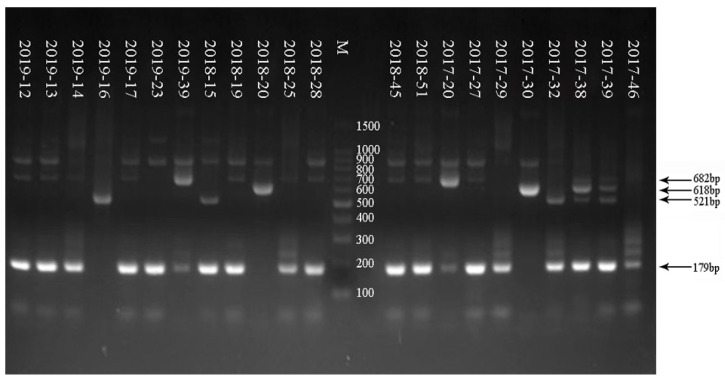
Electrophoretic results of Rotavirus G genotyping in some samples from 2017 to 2019.

**Figure 2:**
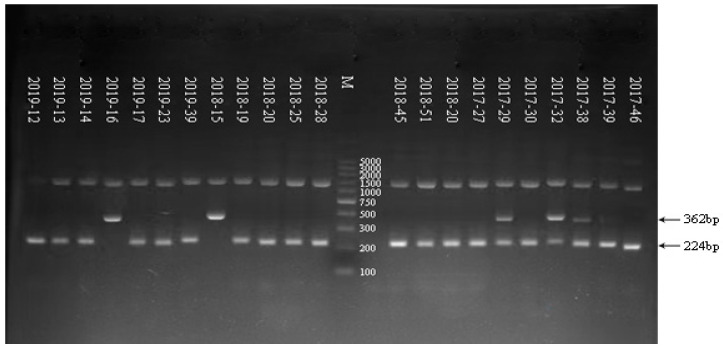
Electrophoretic results of Rotavirus P genotyping in some samples from 2017 to 2019.

As shown in Figure 1, a 100-bp marker was used in the electrophoresis. The G genoserotype was determined according to the electrophoretogram of the G genoserotype of the RV-positive samples from 2017 to 2019. RV-positive samples showed multiple serotypes, including G9, G1, G2, and G3. As shown in Figure 2, a 100-bp marker was used in the electrophoresis. The P genoserotype was determined as P[4] and P[8], respectively, according to the electrophoretogram of the P genoserotype of the RV-positive samples from 2017 to 2019. The details of the G/P genotyping of the samples are shown in Table 5.

### 3.4. Result of G/P Genotyping of RV in 2017

In total, 143 samples from 2017 identified as RV positive by RT-PCR were analyzed for G/P genotyping. Of these, G9 was the dominant genoserotype, accounting for 60.84% (87/143), followed by G2, G1, and G3 accounting for 18.88% (27/143), 9.09% (13/143), and 4.19% (6/143), respectively. The G mixed genoserotype and nonserotype were 4.90% (7/143) and 2.10% (3/143), respectively. The P genoserotype was dominated by the P[8] serotype, accounting for 75.52% (108/143); the P[4] serotype accounted for 16.08% (23/143), mixed serotype accounted for 2.80% (4/143), and the P nonserotype accounted for 5.59% (8/143). The G/P combination was dominated by G9P[8], accounting for 60.84% (87/143), followed by G2P[4], accounting for 10.49% (15/143).

### 3.5. Result of G/P Genotyping of RV in 2018

A total of 207 samples from 2018 identified as RV positive by RT-PCR were analyzed for G/P genotyping. Of these, G9 was the dominant genoserotype, accounting for 95.65% (198/207), followed by G2 and G nonserotypes, which were 2.90% (6/207) and 1.40% (3/207), respectively. The G mixed genoserotype and nonserotype were 4.90% (7/143) and 2.10% (3/143), respectively. The P genoserotype was dominated by the P[8] serotype, accounting for 94.69% (196/207); the P[4] serotype accounted for 3.38% (7/207), and the P nonserotype accounted for 1.93% (4/207). The G/P combination was dominated by G9P[8], accounting for 94.69% (196/207).

### 3.6. Result of G/P Genotyping of RV in 2019

A total of 117 samples from 2019 identified as RV positive by RT-PCR were analyzed for G/P genotyping. Of these, G9 was the dominant genoserotype, accounting for 83.76% (98/117), followed by G3 and G nonserotypes, which were 5.13% (6/117) and 11.11% (13/117), respectively. The P genoserotype was dominated by the P[8] serotype, accounting for 88.89% (104/117); the P[4] serotype accounted for 6.84% (8/117), and the P nonserotype accounted for 4.27% (5/117). The G/P combination was dominated by G9P[8], accounting for 83.76% (98/117), followed by G3P[8], accounting for 5.13% (6/117).

### 3.7. Comprehensive Analysis of G/P Genotyping of RV from 2017 to 2019

**Figure 3:**
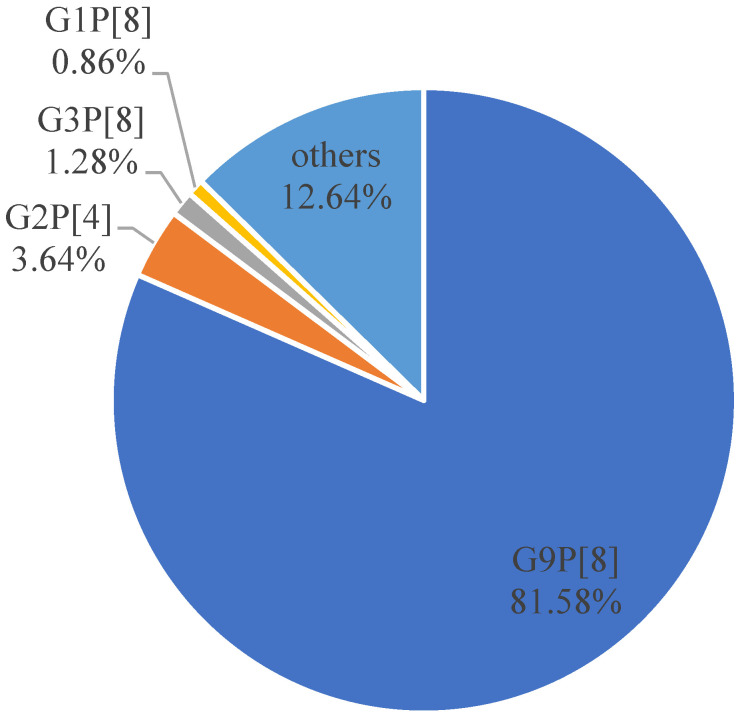
Composition ratio of rotavirus genoserotypes in Yantai in 2017–2019.

The G/P typing result of 467 RV-positive samples from 2017 to 2019 were comprehensively analyzed (Figure 3). The result showed that G9 was the dominant serotype in the G genoserotype, accounting for 82.01% (383/467), followed by G27.07% (33/467), G1 2.78% (13/467), G3 2.57% (12/467), and the G nonserotype 5.57% (26/467). The dominant genoserotypes of P were P[8] serotype, accounting for 83.37% (408/467), the P[4] serotype, accounting for 8.99% (42/467), and the P nonserotype, accounting for 3.64% (17/467). The G/P combination was dominated by G9P[8], accounting for 81.58% (381/467), followed by G2P[4], accounting for 3.64% (17/467), G3P[8], accounting for 1.28% (6/467), and G1P[8], accounting for 0.86% (4/467).

## 4. Discussion

Yantai is located in the northeast of the Shandong Peninsula, near the Bohai Sea and the Yellow Sea, on the “magical latitude” line of 37 degrees north latitude. The epidemic of diseases has a unique pattern. The incidence of diarrhea in Yantai is much higher than that in neighboring cities. In recent years, a lack of a systematic analysis of the pathogenic spectrum, as well as the genotype and genetic variation of infectious diarrhea (containing both viral and bacterial pathogens) covering the whole area of Yantai, has made it difficult to achieve precise prevention and control. RV accounted for the largest proportion of pathogens causing infectious diarrhea in Yantai from 2017 to 2019. The purpose of this study was to conduct systematic surveillance of rotavirus in Yantai City, Shandong Province, from 2017 to 2019, to clarify its epidemiological characteristics, etiology, and pathogen composition, as well as to fill the research gaps in this area, explore and develop targeted prevention and control policies for rotavirus in this region, and play a positive exemplary role for the surrounding municipalities.

From the monitoring results of Yantai City obtained over three consecutive years, we found that the distribution of rotavirus has a certain seasonal pattern, showing obvious seasonality, especially in the winter and spring peaks; therefore, focus on strengthening the prevention and control of rotavirus should prioritize the winter and spring seasons. The incidence occurs mainly in infants and children < 5 years old, and the incidence is highest in the age group of 0 to 5 years old. The immune system is immature in infants and toddlers, and the gastrointestinal function gradually improves with age, therefore the incidence gradually decreases after 5 years of age.

RV can cause rotavirus gastroenteritis (RVGE) by directly attacking the mature epithelial cells in the top and middle of the small intestinal villi, mostly in children under 5 years of age, with the main manifestations being vomiting (typical), diarrhea (watery stools), dehydration, electrolyte disturbances, and, in some cases, low-grade fever. Moreover, it is a global public health problem, and is the leading cause of severe diarrhea in children under 5 years of age in China. Abdominal rotavirus is an important agent of infectious diarrhea in adults and children, especially in infants and young children. RV is currently the leading cause of diarrhea-related deaths worldwide in children under 5 years. The results of our study also showed that infants and children aged 0–5 years accounted for 69.25% of the total rotavirus-infected population, which is the population that needs to be focused on and given priority in prevention and control. The most common G genotypes in humans are G1, G2, G3, G4, G9 and G12.G3, G4, G9, and G12, and the most common P genotypes are P[4], P[6], and P[8]. G2P[4], G3P[8], G4P[8], G9P[8], and G12P[8] are responsible for more than 90% of human RVAs worldwide and have caused more than 90% of RVA-associated diarrhea cases in humans worldwide [17,18]. Although the rate of RV infection has decreased over the past decade, it still poses a heavy disease burden. The development and implementation of effective vaccines are one of the best strategies to reduce the global burden of the disease [13]. RV vaccines have been proven to prevent severe diarrhea [19]. Thus, China is expected to incorporate them into the immunization program as soon as possible.

The epidemic strains differ in different regions. Studies have shown significant relative changes in the prevalence of RV serotypes that require continuous monitoring and determination of trends and prevalence, especially in the post-vaccination period [20]. RV genoserotypes change over time, and some show periodic cycles [21]. Therefore, RV genotyping and the continuous monitoring of emerging strains are critical to understanding the effectiveness of new RV vaccines. Tracking the change trend of RV serotypes and their possible infection source in the region is of high value and significance for disease prevention and control and the provision of vaccine selection.

RV still has the highest proportion of pathogens causing infectious diarrhea in Yantai. The VP7 (G serotype) and VP4 (P serotype) genes of 467 RV-positive samples collected from 2017 to 2019 were amplified by two-round nested RT-PCR for G/P genotyping for better prevention and control. The result showed that G9P[8] RV was the dominant serotype in Yantai for three consecutive years. In 2017, G9P[8] was the predominant serotype accounting for 60.84%; other serotypes also existed, presenting diversification. However, the results in 2018 and 2019 revealed that the rotavirus serotypes in Yantai were relatively single. The proportion of G9P[8] increased significantly compared with that in 2017, accounting for 94.69% and 83.76%, respectively. The reasons might be varied. This is consistent with the prevalent form in most parts of the country. Starting in 2012, G9P[8] became the predominant genotype [22]. In 2017–2018, G9P[8] predominated in Shanghai, accounting for 67.96% [23], and 19. 19% of 961 children under 10 years old in Shanxi Province from 2015 to 2019 were positive for RVA; G9P[8] was the most common (76. 0%) [24]. In recent years, the G9P[8] genotype has become the most predominant genotype in China. Long-term monitoring is still required to determine if this genotype remains prevalent.

The use of an RV vaccine is the most economical and practical approach to prevent RV infectious diarrhea [25,26,27]. The World Health Organization (WHO) issued an enhanced measure in 2009 to support RV vaccination for infants. In 2009, RV vaccination was supported for infants in all regions of the world. RV vaccines should be made available to infants in all regions of the world, especially those with high mortality rates associated with diarrhea. The 2013 WHO position paper on rotavirus vaccines also stated that the use of rotavirus vaccines should be part of a comprehensive strategy to control diarrhea [28], along with preventive measures (such as the promotion of basic hygiene measures, improved water supply and sanitation). Moreover, vaccine use should be part of a comprehensive strategy to control diarrheal diseases. Package (e.g., oral rehydration therapy) RV vaccines have contributed significantly to a global reduction in RV-related diseases for more than a decade. However, RV was still responsible for 128,500 (95%) deaths due to varying RV vaccine coverage in different regions. In 2016, RVS continued to cause deaths among children under 5 years of age, almost all of which occurred in low- and middle-income countries [13]. There are currently two oral rotavirus vaccines available for babies in mainland China, namely the live oral rotavirus vaccine produced by Lanzhou Biologicals in China and the live oral pentavalent recombination rotavirus attenuated vaccine produced by Merck Sharp & Dohme in the United States. The live rotavirus vaccine produced by Lanzhou Biologicals in China is mainly used to prevent diarrhea in infants and children caused by the rotavirus group A rotavirus. The live attenuated rotavirus vaccine produced by Merck Sharp & Dohme in the United States is mainly used to prevent gastroenteritis in infants and children caused by RVA (G1, G2, G3, G4 and G9) and provides 1 year of immune protection. Thus, children younger than 3 years are recommended to be immunized once a year to achieve a better immune effect. RV vaccines have good preventive effects against different RV serotypes. Hence, the results may play a positive role in the selection and use of RV vaccines and disease prevention and control in Yantai. Our results suggested that the oral rotavirus attenuated live vaccine containing the G9 serotype should be the priority vaccination for infants in Yantai in recent years. This result provided precise guidance for vaccine selection. The vaccine is the most effective approach to reduce the RV infection rate. Therefore, relevant departments should actively advocate RV vaccination for children younger than 5 years, strengthen the management of kindergartens and schools, pay attention to food hygiene and eating habits [29,30,31], and reduce the incidence and economic burden induced by diarrhea.

Rotaviruses are constantly evolving, and the use of RV vaccines, changes in strain virulence, changes in receptor type, and the selection of the overall population’s immune pressure’s influence on epidemiological trends [32,33] need to be kept under epidemiological surveillance, while whole-genome sequencing can provide more effective information on genotypic evolutionary pressure and antigenic diversity after vaccination. At the same time, it is critical to study how vaccine-derived immunity influences the evolution and spread of rotavirus, all of which require future exploration [34,35,36]. In this study, the epidemiological characteristics of RV infection and the development of dominant genoserotypes in Yantai were investigated, providing molecular epidemiological information for the prevention and control of diarrhea induced by RV.

Government support for vaccination is essential to ensure mass immunization. Therefore, the development of a safe and effective RVA vaccine is an important tool to prevent infection. Moreover, it is essential to understand the distribution of RVA genotypes for targeted research and the development of RVA vaccines [37,38]. Most importantly, research on RV serotype should be further strengthened to identify locally dominant serotypes and reduce the incidence, economic burden, and impact of infectious diarrhea on patients, families, and society. The continued surveillance of globally transmitted RVA genotypes will provide important data for epidemiological and genotypic diversity [39,40] and inform the selection of appropriate genotypes for current and future vaccine development.

There are some limitations in this study. In the future, we will try to collect information on the epidemiological characteristics of rotavirus in a large sample and analyze the clinical symptoms in relation to the infection type of the rotavirus, especially focusing on the infection type of severe cases, so as to provide some basis for the prevention and treatment of rotavirus in Yantai.

## Figures and Tables

**Table 1:** VP7 RT-PCR Primer.

Primer	Sequence 5′-3′	Nucleotide Position (Expected Size cDNA 881 bp)
VP7F	ATG TAT GGT ATT GAA TAT ACC AC	51–71
VP7R	AAC TTG CCA CCA TTT TTT CC	932–914

**Table 2:** VP4 RT-PCR Primer.

Primer	Sequence 5′-3′	Nucleotide Position (Expected Size cDNA is 663 bp)
VP4F	TAT GCT CCA GTN AAT TGG	132–149
VP4R	ATT GCA TTT CTT TCC ATA ATG	775–795

**Table 3:** G-Typing PCR Primer.

Type	Primer	Sequence 5′-3′	Nucleotide Position	Size PCR Product (bp)
G1	aBT1	CAA GTA CTC AAA TCA ATG ATG G	314–335	618
G2	aCT2	CAA TGA TAT TAA CAC ATT TTC TGT G	411–435	521
G3	G3	ACG AAC TCA ACA CGA GAG G	250–269	682
G4	aDT4	CGT TTC TGG TGA GGA GTT G	480–499	452
G8	aAT8	GTC ACA CCA TTT GTA AAT TCG	178–198	754
G9	G9	CTT GAT GTG ACT AYA AAT AC	757–776	179

**Table 4:** P-Typing PCR Primer.

Type	Primer	Sequence 5′-3′	Nucleotide Position	Size PCR Product (bp)
P[4]	2T-1	CTA TTG TTA GAG GTT AGA GTC	492–474	362
P[6]	3T-1	TGT TGA TTA GTT GGA TTC AA	278–259	146
P[8]	1T-1D	TCT ACT GGR TTR ACN TGC	356–339	224
P[9]	4T-1	TGA GAC ATG CAA TTG GAC	402–385	270
P[10]	5T-1	ATC ATA GTT AGT AGT CGG	594–575	462
P[11]	P[11]	GTA AAC ATC CAG AAT GTG	323–305	191

**Table 5:** Electrophoresis results of G/P typing of rotavirus from 2017 to 2019.

Sample	G Serotype	Size PCR Product (bp) of G Serotype	P Serotype	Size PCR Product (bp) of P Serotype
2019–12	G9	179	P[8]	224
2019–13	G9	179	P[8]	224
2019–14	G9	179	P[8]	224
2019–16	G2	521	P[4]	362
2019–17	G9	179	P[8]	224
2019–23	G9	179	P[8]	224
2019–39	G3	682	P[8]	224
2018–15	G2 + G9	521 + 179	P[4]	362
2018–19	G9	179	P[8]	224
2018–20	G1	618	P[8]	224
2018–25	G9	179	P[8]	224
2018–28	G9	179	P[8]	224
2018–39	G9	179	P[8]	224
2018–45	G9	179	P[8]	224
2018–51	G9	179	P[8]	224
2017–20	G3	682	P[8]	224
2017–27	G9	179	P[8]	224
2017–29	G9	179	P[4] + P[8]	362 + 224
2017–30	G1	618	P[8]	224
2017–32	G2 + G9	521 + 179	P[4] + P[8]	362 + 224
2017–38	G1 + G9	618 + 179	P[4] + P[8]	362 + 224
2017–39	G9	179	P[4] + P[8]	362 + 224
2017–46	G9	179	P[8]	224

## Data Availability

All published data of this study are true and valid, and some data are not published because of patient privacy.

## References

[1] Estes M.K., Greenberg H.B. (2013). Rotaviruses.

[2] Chen J.Z., Settembre E.C., Aoki S.T., Zhang X., Bellamy A.R., Dormitzer P.R., Harrison S.C., Grigorieff N. (2009). Molecular interactions in rotavirus assembly and uncoating seen by high-resolution cryo-EM. Proc. Natl. Acad. Sci. USA.

[3] Li Z., Baker M.L., Jiang W., Estes M.K., Prasad B.V. (2009). Rotavirus architecture at subnanometer resolution. J. Virol..

[4] Settembre E.C., Chen J.Z., Dormitzer P.R., Grigorieff N., Harrison S.C. (2011). Atomic model of an infectious rotavirus particle. EMBO J..

[5] Kirkwood C.D. (2010). Genetic and antigenic diversity of human rotaviruses: Potential impact on vaccination programs. J. Infect. Dis..

[6] RCWG List of Accepted Genoserotype. [EB/OL]. https://rega.kuleuven.be/cev/viralmetagenomics/virus-classification/rcwg.

[7] Troeger C., Forouzanfar M., Rao P.C., Khalil I., Brown A., Reiner R.C., Fullman N., Thompson R.L., Abajobir A., Ahmed M. (2017). Estimates of global, regional, and national morbidity, mortality, and aetiologies of diarrhoeal diseases: A systematic analysis for the Global Burden of Disease Study 2015. Lancet Infect. Dis..

[8] Dodet B., Heseltine E., Saliou P. (1997). Rotaviruses in human and veterinary medicine. Sante.

[9] World Health Organization (2007). Rotavirus vaccines. Relev. Epidemiol. Hebd..

[10] Global Rotavirus Surveillance Network and Rotavirus Age Study Collaborators (2019). Global Review of the Age Distribution of Rotavirus Disease in Children Aged <5 Years before the Introduction of Rotavirus Vaccination. Clin. Infect. Dis..

[11] Isanaka S., Langendorf C., McNeal M.M., Meyer N., Plikaytis B., Garba S., Sayinzoga-Makombe N., Soumana I., Guindo O., Makarimi R. (2021). Rotavirus Vaccine Efficacy up to 2 Years of Age and against Diverse Circulating Rotavirus Strains in Niger: Extended Follow-up of a Randomized Controlled Trial. PLoS Med..

[12] Bergman H., Henschke N., Hungerford D., Pitan F., Ndwandwe D., Cunliffe N., Soares-Weiser K. (2021). Vaccines for PreventingRotavirus Diarrhoea: Vaccines in Use. Cochrane Database Syst. Rev..

[13] Tate J.E., Burton A.H., Boschi-Pinto C., Parashar U.D., Agocs M., Serhan F., de Oliveira L., Mwenda J.M., Mihigo R., World Health Organization–Coordinated Global Rotavirus Surveillance Network (2016). Global, Regional, and National Estimates of Rotavirus Mortality in Children <5 Years of Age, 2000–2013. Clin. Infect. Dis..

[14] Ministry of Health of the People’s Republic of China (2007). WS 271- 2007 Diagnostic Criteria for Infections Diarrhea.

[15] Li J., Pan H., Xiao W.J., Gong X.H., Zhuang Y., Kuang X.Z., Wu H.Y., Yuan Z.A. (2017). Epidemiological and etiological surveillance study of infectious diarrhea in Shanghai in 2013–2015. Chin. J. Prev. Med..

[16] Iturriza-Gómara M., Green J., Brown D.W., Desselberger U., Gray J.J. (2000). Diversity within the VP4 gene of rotavirus P[8] strains: Implications for reverse transcription-PCR genotyping. J. Clin. Microbiol..

[17] Matthijnssens J., Van Ranst M. (2012). Genotype constellation and evolution of group A rotaviruses infecting humans. Curr. Opin. Virol..

[18] Bányai K., László B., Duque J., Steele A.D., Nelson E.A.S., Gentsch J.R., Parashar U.D. (2012). Systematic review of regional and temporal trends in global rotavirus strain diversity in the pre rotavirus vaccine era: Insights for understanding the impact ofrotavirus vaccination programs. Vaccine.

[19] Walker C.L.F., Rudan I., Liu L., Nair H., Theodoratou E., Bhutta Z.A., O’Brien K.L., Campbell H., Black R.E. (2013). Global burden of childhood pneumonia and diarrhoea. Lancet.

[20] Malik A., Haldar P., Ray A., Shet A., Kapuria B., Bhadana S., Santosham M., Ghosh R.S., Steinglass R., Kumar R. (2019). Introducing Rotavirus Vaccine in the Universal Immunization Programme in India: From Evidence to Policy to Implementation. Vaccine.

[21] Wang F., Jin M., Li D., Zhang Q., Li Y. (2019). Molecular epidemiological analysis of viral diarrhea in children under 5 years in Lanzhou in 2017. Chin. J. Biol..

[22] Tian Y., Gao Z., Li W., Liu B., Chen Y., Jia L., Yan H., Wang Q. (2021). Group A rotavirus prevalence and genotypes among adult outpatients with diarrhea in Beijing, China, 2011–2018. J. Med. Virol..

[23] Kuang X., Gong X., Zhang X., Pan H., Teng Z. (2020). Genetic diversity of group A rotavirus in acute gastroenteritis outpatients in Shanghai from2017 to 2018. BMC Infect. Dis..

[24] Zhao L., Shi X., Meng D., Guo J., Li Y., Liang L., Guo X., Tao R., Zhang X., Gao R. (2021). Prevalence and genotype distribution of group A rotavirus circulating in Shanxi province, China during 2015–2019. BMC Infect. Dis..

[25] Isanaka S., Garba S., Plikaytis B., McNeal M.M., Guindo O., Langendorf C., Adehossi E., Ciglenecki I., Grais R.F. (2021). Immunogenicityof an Oral Rotavirus Vaccine Administered with Prenatal Nutritional Support in Niger: A Cluster Randomized Clinical Trial. PLoS Med..

[26] Soares-Weiser K., Bergman H., Henschke N., Pitan F., Cunliffe N. (2019). Vaccines for Preventing Rotavirus Diarrhoea: Vaccines in Use. Cochrane Database Syst. Rev..

[27] Khagayi S., Omore R., Otieno G.P., Ogwel B., Ochieng J.B., Juma J., Apondi E., Bigogo G., Onyango C., Ngama M. (2020). Effectiveness of Monovalent Rotavirus Vaccine against Hospitalization with Acute Rotavirus Gastroenteritis in Kenyan Children. Clin. Infect. Dis..

[28] Troeger C., Khalil I.A., Rao P.C., Cao S., Blacker B.F., Ahmed T. (2018). Rotavirus vaccination and the global burden of rotavirus diarrhea among children younger than 5 years. JAMA Pediatr..

[29] Zhou Y., Zhu X., Hou H., Lu Y., Yu J., Mao L., Mao L., Sun Z. (2018). Characteristics of diarrheagenic Escherichia coli among children under 5 years of age with acute diarrhea: A hospital based study. BMC Infect. Dis..

[30] Zeng M., Chen J., Gong S.T., Xu X.H., Zhu C.M., Zhu Q.R. (2010). Epidemiological surveillance of Norovirus and rotavirus diarrhea among outpatient children in five metropolitan cities. Chin. J. Pediatr..

[31] Mizan M.F.R., Jahid I.K., Kim M., Lee K.H., Kim T.J., Ha S.D. (2016). Variability in biofilm formation correlates with hydrophobicity and quorum sensing among Vibrio parahaemolyticus isolates from food contact surfaces and the distribution of the genes involved in biofilm formation. Biofouling.

[32] Merten S., Schaetti C., Manianga C., Lapika B., Hutubessy R., Chaignat C.L., Weiss M. (2013). Sociocultural determinants of anticipated vaccine acceptance for acute watery diarrhea in early childhood in Katanga Province, Democratic Republic of Congo. Am. J. Trop. Med. Hyg..

[33] Salami A., Fakih H., Chakkour M., Salloum L., Bahmad H.F., Ghssein G. (2019). Prevalence, risk factors and seasonal variations of different Enteropathogens in Lebanesehospitalized children with acute gastroenteritis. BMC Pediatr..

[34] Ali Z., Harastani H., Hammadi M., Reslan L., Ghanem S., Hajar F., Sabra A., Haidar A., Inati A., Rajab M. (2016). Rotavirus Genotypes and Vaccine Effectiveness from a Sentinel, Hospital-Based, Surveillance Study for Three Consecutive Rotavirus Seasons in Lebanon. PLoS ONE.

[35] Zaraket R., Salami A., Bahmad M., El Roz A., Khalaf B., Ghssein G., Bahmad H.F. (2020). Prevalence, risk factors, and clinical characteristics of rotavirus and adenovirus among Lebanese hospitalized children with acute gastroenteritis. Heliyon.

[36] Burnett E., Parashar U.D., Tate J.E. (2020). Real-World Effectiveness of Rotavirus Vaccines, 2006–2019: A Literature Review and Meta-Analysis. Lancet Glob. Health.

[37] Debellut F., Jaber S., Bouzya Y., Sabbah J., Barham M., Abu-Awwad F., Hjaija D., Ramlawi A., Pecenka C., Clark A. (2020). Introduction of Rotavirus Vaccination in Palestine: An Evaluation of the Costs, Impact, and Cost-Effectiveness of ROTARIX andROTAVAC. PLoS ONE.

[38] Lee B. (2021). Update on Rotavirus Vaccine Underperformance in Low- to Middle-Income Countries and next-Generation Vaccines. Hum. Vaccines Immunother..

[39] Ghssein G., Salami A., Salloum L., Chedid P., Joumaa W.H., Fakih H. (2018). Surveillance Study of Acute Gastroenteritis Etiologies in Hospitalized Children in South Lebanon(SAGE study). Pediatr. Gastroenterol. Hepatol. Nutr..

[40] Ibrahim J.N., Eghnatios E., El Roz A., Fardoun T., Ghssein G. (2019). Prevalence, antimicrobial resistance and risk factors for campylobacteriosis in Lebanon. J. Infect. Dev. Ctries..

